# Use of the Medicool™ cooling system to increase efficacy of therapeutic hypothermia post cardiac arrest

**DOI:** 10.1186/cc9726

**Published:** 2011-03-11

**Authors:** I Goodhart, R Porter, A Temple

**Affiliations:** 1 Sheffield Teaching Hospitals NHS Trust, Sheffield, UK

## Introduction

Patients admitted to intensive care (ITU) at Sheffield Teaching Hospitals who have had a cardiac arrest are cooled according to the local therapeutic hypothermia (TH) protocol regardless of rhythm or location of arrest [1]. A previous audit identified poor efficacy in cooling patients to target [2]. Following this, the Medicool™ device was purchased to improve cooling. This aim of this evaluation is to assess the efficacy of cooling with Medicool™.

## Methods

Following local audit committee approval, patients admitted between May 2008 and July 2010 were retrospectively identified from ITU admission records. The following data were collected: demographics, arrest and admission characteristics, details of TH and outcome. Previous audit data from 2008 were also examined [2].

## Results

**Figure 1:**
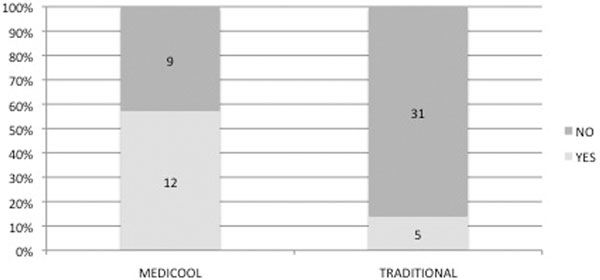
**Patients in whom the target temperature was maintained for 24 hours (*P *= 0.006)**.

Sixty-five patents were admitted to the ITU following cardiac arrest between May 2008 and July 2010. The median age was 67 years (29 to 81), 66% were male. Fifty-two per cent survived to hospital discharge. Forty-eight patients were eligible for cooling; in 43 cooling was performed: 26 were cooled using Medicool™ and 17 using traditional techniques. The median time to reach the target temperature was 4 hours with Medicool™ and 5 hours with traditional techniques. In six patients, cooling was abandoned. In patients who completed 24 hours of cooling, 57% of the Medicool™ patients and 31% of the traditionally cooled patients remained in the target temperature for the entire 24 hours. No patients (*n *= 20) in the previous audit were maintained within the target temperature for 24 hours using traditional techniques. See Figure 1.

## Conclusions

The Medicool™ system increases both the cooling rate and the efficacy of cooling in patients undergoing TH. We would advocate the use of Medicool™ over the traditional cooling techniques. It is more effective and additionally when compared with other more invasive cooling techniques is cheaper to instigate, easy for healthcare professionals to use and is associated with less side effects.
